# Remote, asynchronous training and feedback enables development of neurodynamic skills in physiotherapy students

**DOI:** 10.1186/s12909-023-04229-w

**Published:** 2023-04-20

**Authors:** Ignacio Villagrán, Francisca Rammsy, Javiera Del Valle, Sofía Gregorio de las Heras, Liliana Pozo, Patricio García, Gustavo Torres, Julián Varas, Allison Mandrusiak, Marcia Corvetto, Javiera Fuentes-Cimma

**Affiliations:** 1 Carrera de Kinesiología, Departamento de Ciencias de la Salud, Facultad de Medicina, Pontificia Universidad Católica de Chile, Vicuña Mackenna 4860, Santiago, Chile; 2 Centro de Simulación y Cirugía experimental, Facultad de Medicina, Pontificia Universidad Católica de Chile, Santiago, Chile; 3 School of Health and Rehabilitation Sciences, The University of Queensland, Brisbane, Australia; 4 School of Health Professions Education (SHE), Maastricht University, Maastricht, the Netherlands

**Keywords:** Distance learning, Formative feedback, Clinical skills, Physiotherapy, Musculoskeletal manipulations

## Abstract

**Background:**

During the COVID-19 pandemic, face-to-face teaching and learning of physiotherapy practical skills was limited. Asynchronous, remote training has been effective in development of clinical skills in some health professions. This study aimed to determine the effect of remote, asynchronous training and feedback on development of neurodynamic skills in physiotherapy students.

**Methods:**

Longitudinal repeated measurements study, across four training sessions. Participants engaged in a remote training program for development of upper limb neurodynamic techniques. In this sequential training, participants viewed the online tutorial, practiced independently, and uploaded a video of their performance for formative assessment and feedback from a trained instructor via a checklist and rubric.

**Results:**

Intra-subject analyses of 60 third-year physiotherapy students showed that the target standard of performance, with no further significant change in scores, was attained following session 2 for the checklist and session 3 for the rubric. This shows that two sessions are required to learn the procedures, and three sessions yield further improvements in performance quality.

**Conclusion:**

The remote, asynchronous training and feedback model proved to be an effective strategy for students’ development of neurodynamic testing skills and forms a viable alternative to in-person training. This study contributes to the future of acquiring physiotherapy clinical competencies when distance or hybrid practice is required.

**Supplementary Information:**

The online version contains supplementary material available at 10.1186/s12909-023-04229-w.

## Background

The mastery of practical skills is a fundamental step in the development of clinical skills [1]. To facilitate the attainment of practical skills, students engage in progressive learning methodologies that include repeated supervised practice and clinical simulation [2]. However, due to the COVID-19 pandemic, educators have needed to adapt, rapidly, to integrate online methodologies [3–6] meaning a pivot away from traditional face-to-face approaches and towards remote training models.

The concept of remote training proposed in this study is based on the definition of remote simulation, which refers to “simulation performed with the instructor, students, or both in different locations to complete educational or evaluation activities, which can be synchronous or asynchronous” [7]. Remote, asynchronous simulation (where the learner works through the content and practices skills in their own time), has been implemented with some limitations, however, it was found that allow the training of medical skills and procedures, being effective for training and assessment of laparoscopy skills [8] and critical care skills [9]. While it has been reported that online videos could be an effective method of instruction for psychomotor skills in some areas of musculoskeletal physiotherapy [10], to our knowledge, sequential asynchronous strategies with feedback in an online platform have not been explored in physiotherapy training.

Feedback on practical skills acquisition is an integral part of the learning process; it allows the student to reflect on their actions and decisions, with the aim of encouraging the transfer of knowledge, skills, and behavior, which is fundamental to learning and reducing errors in clinical practice [11]. Feedback requires expert instructors capable of providing objective information about student performance, however this can be resource intensive and dependent on teacher availability at a set time [12]. A key benefit of remote, asynchronous training is that it allows for the delivery of feedback to students at remote locations, without requiring a classroom or synchronous instructor supervision [8, 9, 13]. In addition, this model allows the student to practice the desired skills, independently, with feedback, until reaching a certain level of learning or target standard of performance.

Remote, asynchronous training has been implemented in the context of practical techniques that require a learning curve and benefit from personalized feedback [14, 15]. This strategy typically includes the following four steps: (i) students watch a video tutorial of the intended skill, (ii) students practice this skill autonomously, (iii) students record a video of their performance of the skill and upload it to an online platform, and (iv) an expert provides personalized feedback and assessment of the skill [9]. This remote, asynchronous training model has been shown to maintain engagement of both health professional students and educators during the COVID-19 pandemic in a safe and effective way [9, 13].

World Physiotherapy (2021) determined that at least one third of a physiotherapy curriculum should be based on practice education [16]. The COVID-19 pandemic has had significant impacts on face-to-face teaching and learning and the clinical practice of physiotherapy students globally. Accordingly alternative, innovative approaches are needed [3, 17, 18]. Rooted in the active learning theory [19], the purpose of this study was to determine the effect of a remote, asynchronous training program and feedback on development of practical skills (neurodynamic testing procedures) in undergraduate physiotherapy students. The specific objectives of this study were: (1) To design and implement remote training with asynchronous feedback for the development of neurodynamic skills in physiotherapy students: (2) To determine the learning curve of students’ neurodynamic skills through specific and global assessment instruments; (3) To explore students’ perceptions of the acceptability of the remote training method.

## Methodology

### Design and participants

A longitudinal study design was used, with repeated measurements across four sequential training sessions and with asynchronous feedback between each session. Physiotherapy students enrolled in the *Musculoskeletal physiotherapy assessment course* at Pontificia Universidad Católica de Chile (PUC) were invited to participate. This is a third-year course in a five-year undergraduate physiotherapy program, and focuses on musculoskeletal clinical assessment skills, including neurodynamic techniques. In this course, students are expected to select and perform a musculoskeletal assessment adequately and interpret the results through an appropriate clinical reasoning process.

During our five-year program, the third year is when they start to have more practical and clinical instances. However, given the pandemic context, the students had no previous experience with patients. Regarding previous skills, the students had acquired basic assessment techniques (e.g., goniometry, muscle strength, and flexibility assessment), this activity being their first learning of neurodynamic techniques.

A neurodynamic technique in the context of assessment could be defined as a test with a specific combination of spine and limb movements that apply mechanical forces to a part of the nervous system intending to determine whether a patient’s symptoms are related to increased nerve mechanosensitivity [20]. The clinician, student in this case, must thoroughly understand the procedures and their interpretation to increase the sensitivity and test specificity. Neurodynamic assessment tests require a sequence of movements that place the joints in positions where the assessed nerve is most stressed, looking for the patient’s symptoms. These sequential movements can be complex for students to learn, but they are low risk for the model and patient. The models were not actual patients in the training context, so most did not reproduce real symptoms. Finally, during the technique, the therapist must be in constant communication with the patient, considering the symptom that could appear, obtaining the necessary information, identifying the concordant symptom of the patient, and whether the differentiating movement modifies the symptoms [21].

Whilst engagement in the learning experiences was a compulsory part of the course, participation in the study was voluntary and students provided informed consent. Students were excluded from the study if they did not complete the course within the standard timeframe, or if they were enrolled in the course for a second time (since they had learned neurodynamic skills the previously). The study protocol was reviewed and approved by the Medicine and Health Sciences ethics committee of PUC (Protocol ID: 2,005,514,006).

### Training program

**Fig. 1:**
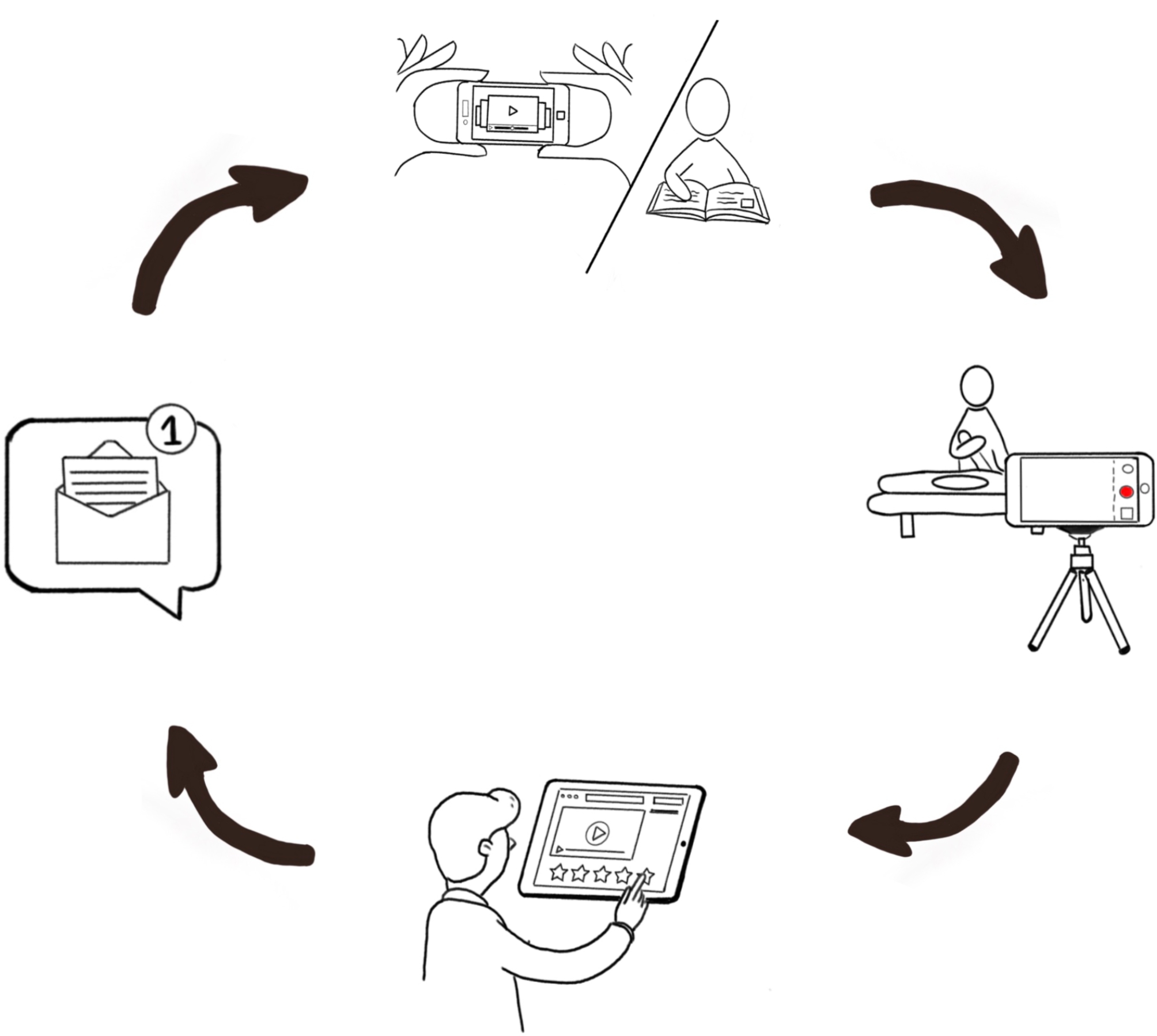
Four-step sequential training process for each session. (1) student watches the videos tutorials, (2) records and uploads the video, (3) instructor views the video, evaluates performance and provides feedback, (4) student receives feedback, practices, and prepares for new delivery

Participants engaged in four sessions of remote, asynchronous training of neurodynamic assessment tests, specifically, the upper limb tension tests (ULTT). They practiced the Upper Limb Tension Test 1 (ULTT1, Median nerve bias), Upper Limb Tension Test 2B (ULTT2B, Radial nerve bias), and the Upper Limb Tension Test 3 (ULTT3, Ulnar nerve bias) in each session of the remote training across five weeks. These assessment techniques are beneficial as complementary information to the sensitive motor and reflex examination. The techniques are well documented in the literature [21–25] and physiotherapy assessment books [20, 26]. This strategy was based on the C1DO1 (“see one, do one”) platform, a locally developed digital tool with a web version and application for iOS and Android [27]. The platform engages students in sequential training and allows instructors to provide multimodal feedback (i.e., written, drawings, audio, or video) on students’ self-recordings. Consequently, students receive feedback on their performance, in a timely, direct, and individualized manner. Each session consisted of a four-step sequential training process (Fig. 1): (1) On the platform, the student watched tutorial videos showing how to perform each technique step by step safely on a simulated patient/model ; (2) following the tutorial video and with the help of a relative or friend posing as a patient/model, the student recorded a video performing the three techniques, consecutively, and uploaded their video onto the C1DO1 platform; (3) the instructor assessed the performance using a rubric, and provided multimodal feedback through the same platform; (4) the student received feedback and formative assessment via the rubric, and continued practicing the neurodynamic techniques until they felt ready to re-record their performance and start the cycle again. This four-step process was repeated across four sessions with the same three neurodynamic techniques.

Between each session, students had five workdays to review the assessment rubric and feedback from the previous session and re-record the three techniques in their new video. The instructors had two workdays to evaluate student videos and provide feedback. At the end of the training program, participants completed an online survey about their experiences and perceptions related to the remote, asynchronous training process.

A purpose-designed observation checklist (Appendix 1) and assessment rubric (Appendix 2) were used by the instructor to evaluate student performance. We developed both instruments based on the steps that compose the techniques and the skills necessary to carry them out with the advice of musculoskeletal-physiotherapist specialists. In addition, the instruments were validated by an expert panel judgment through Delphi method of three rounds. The instruments were available to students and instructors on the C1DO1 platform throughout the training. The instructors recorded the score for each video in a database. The maximum score was 34 points for the checklist and 21 points for the rubric.

Five students from higher years of the physiotherapy program were recruited as instructors and were trained by the teaching team on using the platform and providing multimodal feedback.

### Statistical analysis

Descriptive analyses and intra-subject analyses of variance (ANOVA) with repeated measures were used to quantify the students’ performance during the four training sessions. The descriptive analyses determined the percentage of students who reached the “target standard of performance” in each session, defined as a minimum score of 30 out of 34 points for the checklist (equivalent to 88% of the maximum score) and 19 out of 21 points for the rubric (equivalent to 90% of the maximum score). This target standard of performance was determined by the teaching team to represent optimal and safe performance of each technique.

The data distribution and homogeneity of variances was determined through the Shapiro-Wilk Test and the Mauchly Test, respectively. Post-hoc contrasts identified which videos showed significant differences in student performance considering *p* < 0.05. A descriptive analysis of the student experience survey was performed.

## Results

### Participants

Of a total of 73 students enrolled in the course, 13 students were excluded. One was doing the course for the second time, so he had previously acquired neurodynamic skills, two students suspended their studies, and ten completed the training out of time due to health problems or quarantine related to COVID-19. Finally, data was collected and analyzed for 60 participants who completed the training and uploaded the four videos (total = 240 videos). The participants’ age ranged between 20 and 29 years, and 56.6% were female.

### Descriptive analysis

Each student uploaded one video per stage, with a required of 3 to 10 min as minimum and maximum duration, obtaining an average length of 3 min 44 s. Regarding feedback, in the first video instructors provided an average of 10 feedback inputs (i.e., written comments, audios, and drawings within the students’ videos) to each student, in the second video an average of 7 inputs to each student, in the third video an average of 5 inputs to each student, and in the final video an average of 5 feedback inputs to each student.

**Fig. 2:**
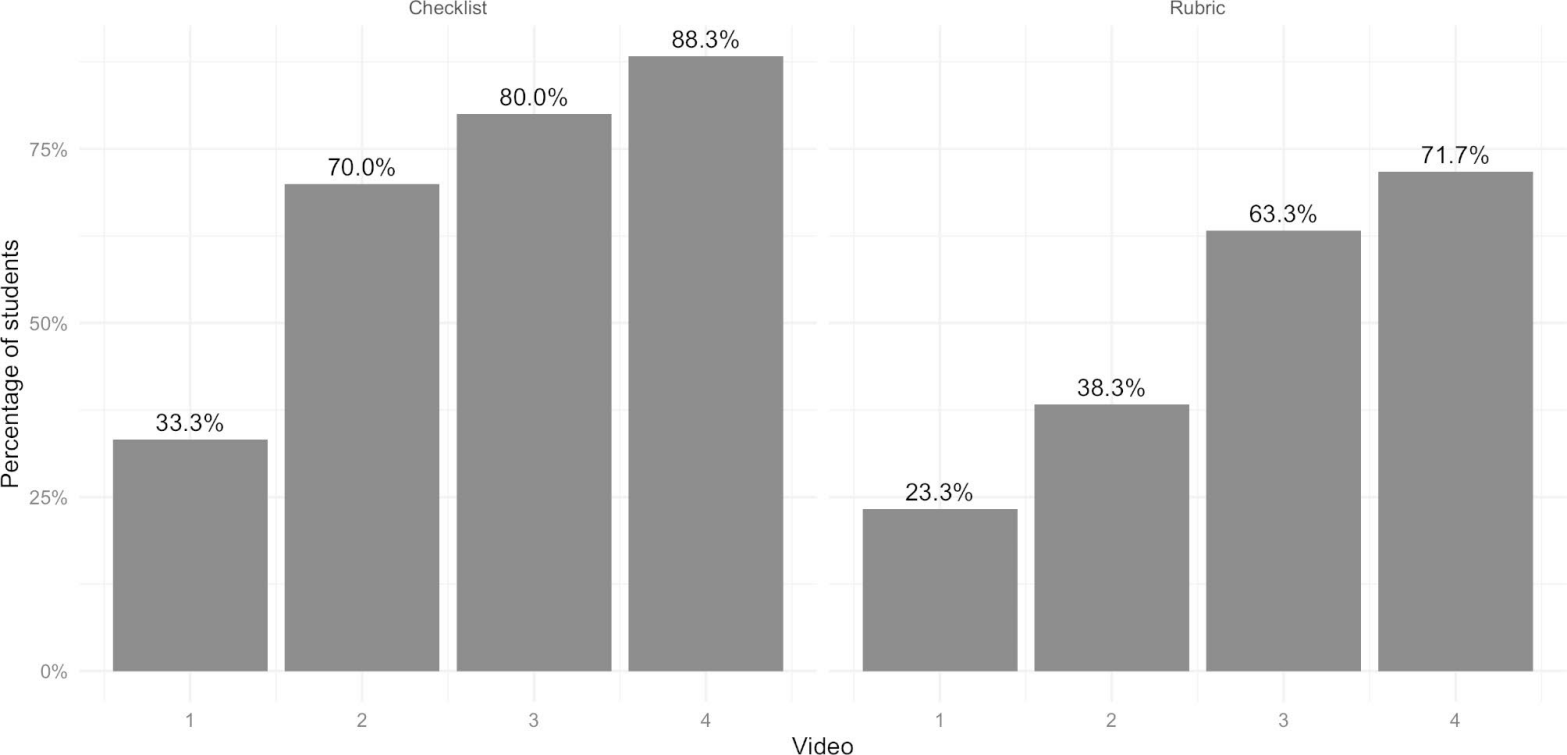
Percentage of students who obtained minimum scores, as defined by the competence criteria, in the measurements evaluated with the checklist and the rubric. The X axis displays the number of training sessions, and the Y axis shows the percentage of minimum or higher scores obtained by the students in each session

Figure 2 shows the percentage of students who obtained the minimum score (i.e., target standard of performance) or higher each session in both the rubric and the checklist. There is a progressive improvement in student performance, shown by the increase in the percentage of students achieving the target standard, on both instruments.

Table 1 shows an increase in the students’ scores from one session to the next, reflected in the increase in mean values and the decrease in standard deviation between deliveries. Hence, as the training progresses, students approach the maximum score.

**Table 1:** Descriptive analysis of the scores obtained from the checklist and rubric

	Checklist (out of 34 points)		Rubric (out of 21 points)	
	Mean	Standard deviation	Mean	Standard deviation
Video 1 Video 2 Video 3 Video 4	28.63 30.88 31.57 32.17	3.19 2.6 2.35 2.26	16.32 17.73 18.85 19.18	2.57 2.46 2.6 2.41

### Verifying assumptions

The Shapiro-Wilk Test determined a normal distribution of the sample for both instruments for each session (*p* < 0.05). To evaluate the homogeneity of variances, the Sphericity Test or Mauchly Test demonstrated, with a 95% confidence, that, for the checklist, the variances between the deliveries were equal. Regarding the rubric, it was demonstrated, with a 95% confidence, that the variances between sessions were not equal and therefore a corrected p-value according to the Sphericity test was used for the ANOVA.

### Session-by-session performance analysis

For the checklist, there were differences in the performance in at least one of the videos compared to the others (*F* (3, 177) = 44.53, *p* < 0.001). For the rubric, because the Mauchly test indicated a violation of sphericity, the degrees of freedom were corrected using the Huynh-Feldt procedure (ε = 0.92). The results showed that, as in the checklist, there were differences in the performance in at least one of the videos compared to the others (*F* (3, 177) = 27.92, *p* < 0.001).

**Fig. 3:**
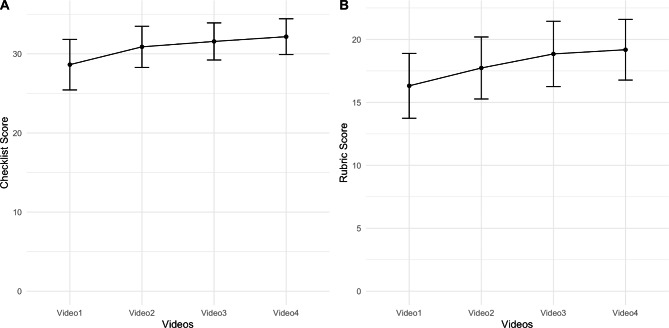
A. Distribution of checklist scores according to the measurements. The X axis shows the number of the video, and the Y axis shows the students’ checklist score in each session (A), and rubric score in each session (B)

The post-hoc contrast for the checklist reported significant differences between Video 1 and 2 (t(59) = -6.67; p < 0.01), between Videos 1 and 3 (t(59) = -8.54; p < 0.001), between Videos 1 and 4 (t(59) = -9.18; p < 0.001), and between Videos 2 and 4 (t(59) = -4.32); p < 0.05). On the other hand, the difference between Videos 2 and 3 was not significant (t(59) = -2.27; p = 0.36), and likewise the difference between Videos 3 and 4 was not significant (t(59) = -2.07; p = 0.56). These results indicate that after session 2 (i.e., Video 2) students do not display a statistically significant difference in performance (Fig. 3A).The post-hoc contrast for the rubric reported significant differences between Videos 1 and 2 (t(59) = -4.25; p < 0.001), between Videos 1 and 3 (t(59) = -6.58; p < 0.001), between Videos 1 and 4 (t(59) = -7.08; p < 0.001), between Videos 2 and 3 (t(59) = -3.45; p < 0.05) and between Videos 2 and 4 (t(59) = -4.39; p < 0.001). On the other hand, the difference between Videos 3 and 4 was not significant (t(59) = -1.16; p = 1). These results indicate that after session 3 (i.e., Video 3) students do not display a statistically significant difference in performance as indicated on the rubric (Fig. 3B).

### Students’ perception of the remote training program

Table 2 shows the students’ perception of their experience with the training program. Most participants agreed or fully agreed that the C1DO1 platform was easy to use (95%) and that the instructions were clear (91.7%). Regarding the training, 65% of the participants reported that they agreed or fully agreed that it allowed them to acquire practical skills, and 88.4% agreed or fully agreed that the feedback helped them to improve across sessions. Concerning confidence, the biggest percentage of participants moved from a perception of “insecurity” (43.3%) in their performance the first time to a perception of “confidence” (41.7%) or “high confidence” (33.3%) in their performance of the techniques after the training. In addition, the 48.3% of the participants stated that they felt prepared to perform the techniques on people with real conditions after completing the training.

**Table 2:** Students’ perception of the training program via asynchronous remote training (n = 60)

Questions	Strongly disagree	Disagree	Neither agree, nor disagree	Agree	Fully agree
The platform is easy to use and navigate	0	3.3	1.7	28.3	66.7
The instructions and supporting information are clear and easy to find	0	3.3	5	45	46.7
The asynchronous remote training methodology allowed me to acquire practical skills	1.7	8.3	25	50	15
The provided feedback helped me to improve from session to session	1.6	3.3	6.7	31.7	56.7

Questions	Very insecure	Insecure	Neither confident, nor insecure	Confident	Very confident
When I first performed the technique, I felt:	25	43.3	25	6.7	0
When I performed the technique the last time, I felt:	1.7	3.3	20	41.7	33.3

Questions	Not prepared	Not very prepared	Neutral	Prepared	Very prepared
After completing the training, how well prepared do you feel to perform the neurodynamic techniques on a real patient?	3.3	11.7	36.7	41.7	6.6
Values expressed in percentages					

## Discussion

The results of this study indicate that a remote, asynchronous training and feedback model is effective in supporting physiotherapy students to develop skills in upper limb neurodynamic techniques. Only two to three training sessions were required to achieve the target standard of performance. These findings contribute important knowledge to physiotherapy education, particularly in the context of the COVID-19 pandemic, which has required rapid transition to remote teaching and learning strategies.

The acquisition of clinical and procedural skills through practice is an essential part of the training of health professionals [28], yet the restrictions imposed by the COVID-19 pandemic revealed challenges when face-to-face practice is not feasible. This study proposed a model of remote teaching and learning of physiotherapy practical skills and determined that it is possible to train neurodynamic skills, remotely.

During the pandemic, some innovative methodologies for training practical skills were described in the literature, such as video recordings of students replicating a physiotherapy technique [29]. Although learning objectives can be achieved, there can be considerable burden on teachers of large courses [29], and synchronous training and feedback can be limited by the teachers’ availability. The proposed model offers asynchronous training that can be self-paced by the student, and feedback provided by the instructor at a suitable time. Further, drawing on a network of instructors enhances capacity to provide individualized feedback, and helps enhance efficiencies in feedback processes [9].

In this investigation, students required two to three sessions to meet the target standard for the neurodynamic skills. Also, as the stages progressed, the number of assigned feedback inputs decreased until a plateau was reached in the last two videos, which could be explained by the fact that the students demonstrated better skills, requiring fewer feedback inputs from the instructor. Although the techniques selected for this study are not considered complex for expert physiotherapists, it should be noted that participants were naïve to these techniques, and these are foundational skills that are a necessary part of the physiotherapist’s toolkit. It is important to note that unlike some other health procedures [8, 13, 30] the skills selected in this area of musculoskeletal physiotherapy typically do not require sophisticated instruments or complex implementation; this low-cost approach facilitates systematic implementation of these types of methodologies in educational programs.

The instruments used in this study were designed to help both students and teachers to structure the performance of techniques and to engage in feedback and assessment. The literature supports the use of checklists for the assessment of practical skills training [31, 32]. Practical techniques tend to be sequential and predictable, so the structure of a checklist allows for a detailed and objective assessment of compliance with the sequence of steps [33]. In a systematic review, it was concluded that further development of valid and reliable instruments for the evaluation of practical skills is needed in physiotherapy [34]; hence, in the absence of “gold standard” instruments, we developed custom measures that proved to be fit for purpose.

Rubrics such as OSATS (Objective Structured Assessment of Technical Skills) have been reported in structured medical-surgical procedures with satisfactory results [30, 35]. Rubrics make it possible to determine the skills necessary to carry out a procedure using satisfaction scales [34]. In our study, the decision to use two instruments was made because, although the checklist is widely used in the literature, it cannot accurately distinguish the level of quality of the student’s practical skills. Therefore, the incorporation of descriptive scales has been suggested to determine the skills necessary to carry out different procedures [36–38]. In the proposed training, students had to ask the patients for symptoms and communicate with them constantly, so it was also essential to obtain information and explain the technique through proper communication skills. We believe that having global and specific instruments offered us options for assessing skills by determining their presence and grading quality. Consequently, different results were obtained from both instruments, since the analysis determined that more sessions are necessary when performance was evaluated using the rubric. We believe that this difference may be due to the further improvement in quality that occurs across sessions (i.e., by the third session) identified by the rubric, beyond attainment of the correct sequence of steps (which was achieved by the second session) identified by the checklist.

According to the perceptions gathered from the participants of this study, remote training of procedural skills using the platform is well accepted by most of the students. In a large proportion, they perceive that it is easy to use, helps to develop practical skills and that the feedback it provides improves skills between sessions. Moreover, this remote training improves the students’ confidence in performing the techniques, decreasing the perceived insecurity in applying the maneuvers.

The impact of the training described in this study can be considered at different organizational levels. At the student level, the consequence of the drastic lifestyle changes due to the pandemic are well-known, with students experiencing high levels of anxiety and depression [39]. The literature reports students’ high regard for hands-on classroom learning and social support from peers and tutors [40, 41] as well as their desire for feedback when learning skills remotely [42]. Our study revealed that students appreciate remote training with asynchronous feedback as it allowed them to continue their studies, from home, and thus acquire practical skills and to improve with practice and feedback. Finally, after completing the remote training, almost half of students reported feeling ready to perform neurodynamic techniques on people with real conditions, but a great percentage of students reported feeling neutral and a small group keeps feeling not ready. Considering that performances scores improved across sessions, it is necessary to compare perceptions results with the transfer of the skills trained to the real setting, which assessment is desirable to prove the effectiveness and real utility of the training program [2]. These positive perceptions about their learning experiences support future directions to combine training modes, including classroom activities and online resources to complement and optimize students’ learning of practical skills [41]. At the teacher level, this model contributes an asynchronous innovative alternative to those published in the literature, for example online discussion of clinical cases [18], use of video podcasting to review practical exams and to encourage the repetitive practice of skills [43], and evaluation based on videos, video conferencing and virtual case-based instruction [29].

### Limitations

This study was conducted at a single university, with three set musculoskeletal techniques, so results may not be transferable across techniques, especially those that differ in their degree of difficulty and materials required. Additionally, the small sample size in a restricted pandemic context may affect the transferability of results regarding students’ perceptions of this strategy. It would have been optimal to add a control group who received traditional training; however, this was not possible in the context of the pandemic.

Regarding the student training conditions, the techniques in this study required a person to perform the techniques on, which may not always be possible (e.g., for students living alone), being a barrier to the optimal implementation of the strategy, so it should be considered in its planning. In addition, we believe that access to the internet and technological devices can also be a barrier. This should be addressed early on with students to support them with the resources needed or flexibility required to complete their training. We think that the asynchronous nature of the model could mitigate this challenge, whereby students could arrange access to a person or technology at a suitable time and practice at their own pace versus at a set, synchronous time.

Another aspect that could be considered a limitation is the workload required for systematic feedback, which can be significant for large classes. However, in our experience, instructors optimized their feedback workload as they became experienced with the platform and its options (e.g., fast-forward video analysis tool) supported by the fact that the students respected the length instructions of the videos. Furthermore, since the feedback was provided asynchronously, instructors could arrange to provide feedback at their convenience. Lastly, the “train the trainer” strategy allowed us to incorporate higher-level students who were trained in the instruction of feedback on the specific procedures, which helped increase capacity and was well-valued by the trainees as an opportunity to develop their feedback skills. This strategy also allows the incorporation of peer feedback. This could contribute to the sustainability of the strategy and provide additional learning opportunities not only for the student receiving the feedback but also for the student giving the feedback through the development of critical thinking and reflective practice [44].

#### Future directions

We believe that this study opens the way for future research to determine the effectiveness of remote, asynchronous training in physiotherapy, but it would be ideal if they consider a control group to determine the effectiveness of the strategy versus conventional face-to-face training and to get a less restricted view of students’ perceptions. To study the effect of this methodology on the acquisition of other techniques or procedures, it is necessary to determine how skills acquired through remote training are transferred to a real context and maintained over time [30]. Furthermore, it is possible that methodologies such as these, where repetition and feedback are the focus of learning, may be more effective in acquiring practical skills. In this regard, investigation of the effect of remote learning compared to traditional in-person and hybrid learning approaches will be helpful, including the time taken to achieve competence, the benefits of the self-paced nature of the model, and the impact on instructor time.

Regarding in-person practice, it is essential to explore how the lessons learned in our study also support face-to-face teaching. In that context, incorporating asynchronous strategies could help mitigate some difficulties of traditional procedural training, delivering asynchronous opportunities to limited synchronous practice times and allowing the learner to receive individualized feedback and demonstrate improved performance prior to summative assessments or real patient encounters. In addition, providing training spaces at the university where students can record themselves would encourage students to practice with the necessary equipment and open up opportunities for peer feedback during the recording process. This could help offload synchronous lectures and encourage the learning of clinical skills that need to be demonstrated with actual patients.

Another opportunity relates to the large amounts of educational data collected from interactions between students and teachers through technology, such as learning analytics, which emerges as an opportunity with great potential to informs the future of higher education [45]. Finally, it appears that technology is here to stay, and results of our technology-based training shapes future advances in health professions education.

## Conclusion

A remote, asynchronous training model is an effective strategy for physiotherapy students’ acquisition of practical skills in upper limb neurodynamic techniques. Through a checklist, it was shown that after the second training session, students were able to reach the target performance level related to correct order of steps. Performance quality, as scored by a rubric, reached the target level by the third training session. The students reported positive perceptions of the remote training, valuing the teacher feedback and usability of the platform, and highlighting an improvement in their confidence and preparation to perform the techniques on people with real conditions. Therefore, remote training platforms with multimodal feedback are a practical solution for attainment of skills when face-to-face teaching and learning is not possible, and there are implications for practice even after the pandemic.

## Electronic supplementary material

Below is the link to the electronic supplementary material.

Supplementary Material 1

Supplementary Material 2

## Data Availability

The datasets generated and analysed during the current study are not publicly available due privacy/ethics reasons but are available from the corresponding author on reasonable request.
