# Supplementary material for: Remote, asynchronous training and feedback enables development of neurodynamic skills in physiotherapy students
Source: BMC Med Educ. 2023 Apr 20;23:267. doi: 10.1186/s12909-023-04229-w (PMC10116106; doi:10.1186/s12909-023-04229-w)
Supplement: Supplementary file 1 — Supplementary Material 1 [file 12909_2023_4229_MOESM1_ESM.pdf]

Appendix – Observation checklist for assessment of neurodynamic techniques of median, ulnar, and radial nerves.

		YES (1 point)	NO (0 points)
	**NEURODYNAMIC TECHNIQUE MEDIAN NERVE**		
1	Positions the patient correctly (supine at edge of bed)		
2	The therapist is in ipsilateral position to the side to be treated, between the patient's thorax and arm, looking at the patient in cranial direction		
3	Performs shoulder fixation with hand in cranial position		
4	Puts the hand in caudal position on the patient's hand with pistol grip, while supporting the patient's elbow on the crest of the therapist's hip flexors		
5	Executes abduction of the glenohumeral joint with elbow flexion		
6	Performs external rotation of the glenohumeral joint		
7	Executes supination of forearm, extension of wrist and fingers (in that order)		
8	Performs elbow extension		
9	Performs lateral neck flexion for differentiation test (if symptoms occur earlier, performs at that time)		
10	Constantly asks the patient about his/her symptoms		
11	Mentions that he/she will perform the technique on the other side		
	**NEURODYNAMIC TECHNIQUE ULNAR NERVE**		
12	Positions the patient correctly (supine at edge of bed)		
13	The therapist is in ipsilateral position to the side to be treated, between the patient's thorax and arm, looking at the patient in cranial direction		
14	Performs shoulder fixation with light depression		
15	Positions his/her caudal hand on the palm of the patient's hand		
16	Extends wrist and fingers		
17	Pronates forearm		
18	Flexes elbow		
19	Performs external rotation of the glenohumeral joint		
20	Executes abduction of the glenohumeral joint with the thigh		

21	Performs lateral neck flexion for differentiation test (if symptoms occur earlier, performs at that time)		
22	Constantly asks the patient about his/her symptoms		
23	Mentions that he/she will perform the technique on the other side		
	**NEURODYNAMIC TECHNIQUE RADIAL NERVE**		
24	Positions the patient correctly (supine at edge of bed)		
25	The therapist positions over the patient’s shoulder, looking in caudal direction in fencing position		
26	Depresses shoulder with his/her hip (ASIS)		
27	Positions the hand flat on the back of the patient's hand and the other hand sideways on the back of the elbow		
28	Performs elbow extension		
29	Performs internal rotation of the glenohumeral joint and pronates the forearm		
30	Flexes wrist and fingers		
31	Performs abduction of glenohumeral joint		
32	Performs lateral neck flexion for differentiation test (if symptoms occur earlier, performs at that time)		
33	Constantly asks the patient about his/her symptoms		
34	Mentions that he/she will perform the technique on the other side		
	**Total Score**		
