# Supplementary material for: Remote, asynchronous training and feedback enables development of neurodynamic skills in physiotherapy students
Source: BMC Med Educ. 2023 Apr 20;23:267. doi: 10.1186/s12909-023-04229-w (PMC10116106; doi:10.1186/s12909-023-04229-w)
Supplement: Supplementary file 2 — Supplementary Material 2 [file 12909_2023_4229_MOESM2_ESM.pdf]

Appendix – Rubric for assessment of neurodynamic techniques of median, ulnar and radial nerve

	Excellent (3)	Good (2)	Regular (1)	Deficient (0)
**Starting position – Patient position**<br>Position the patient and the segment to be assessed according to the description of the assessment technique. With the correct use of the appropriate support elements (towel)	Positions the patient correctly in all 3 techniques	Positions the patient correctly in 2 techniques	Positions the patient correctly in only 1 technique	Patient's position is wrong in all 3 techniques
**Starting position – Therapist position**<br>Therapist adopts the position described for the assessment technique, which favors its execution	Therapist is positioned correctly in all 3 techniques	Therapist is positioned correctly in 2 techniques	Therapist is positioned correctly in only 1 technique	Therapist's position is wrong in all 3 techniques
**Stabilization of the patient during technique execution**<br>Places the stabilizing hand in the correct location according to the description of the assessment technique and stabilizes safely during the technique execution	Stabilizes the patient correctly in all 3 techniques	Stabilizes the patient correctly in 2 techniques	Stabilizes the patient correctly in 1 technique	Stabilization of the patient is wrong in all 3 techniques
**Position of the therapist's hands**<br>Places the mobilising hand in the correct position and mobilises in the right direction according to the description of the assessment technique.	Positions hands correctly in all 3 techniques	Positions hands correctly in 2 techniques	Positions hands correctly in only 1 technique	Position of hands is wrong in all 3 techniques
**Procedure - performs the corresponding steps sequentially**<br>Performs the corresponding steps sequentially according to the description of the assessment technique.	Performs the corresponding steps sequentially in all 3 techniques	Performs the corresponding steps sequentially in 2 techniques	Performs the corresponding steps sequentially in only 1 technique	Does not perform the corresponding steps sequentially in any of the 3 techniques
**Procedure - Shows fluidity when performing the technique (continuity)**<br>Demonstrates fluidity in all the steps according to the description of the assessment technique	Shows fluidity during the execution of all 3 techniques	Shows fluidity during the execution of 2 techniques	Shows fluidity during the execution of only 1 technique	Does not show fluidity in any of the 3 techniques
